# Supplementary material for: Pre-Existing Interstitial Lung Abnormalities Are Independent Risk Factors for Interstitial Lung Disease during Durvalumab Treatment after Chemoradiotherapy in Patients with Locally Advanced Non-Small-Cell Lung Cancer
Source: Cancers (Basel). 2022 Dec 17;14(24):6236. doi: 10.3390/cancers14246236 (PMC9776853; doi:10.3390/cancers14246236)

Supplementary Materials:

Supplementary Figure 1. Examples of ILAs.

These are CT images of ILAs, (A) ground glass attenuation, (B) reticulation, (C) honeycombing, (D) centrilobular nodularity, and (E) traction bronchiectasis.

ILAs, interstitial lung abnormalities; CT, computed tomography

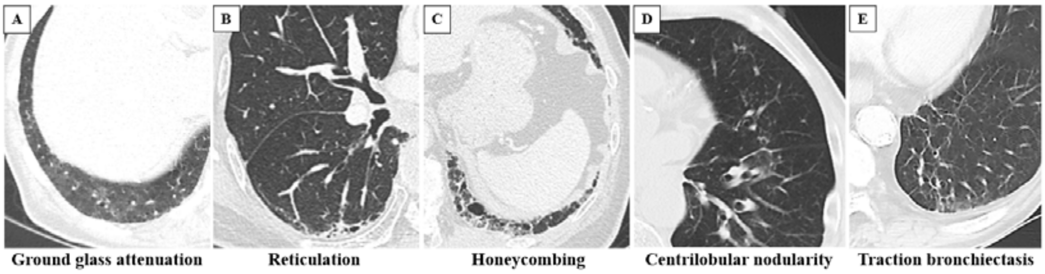

Supplementary Figure 2. Flowchart of patient selection

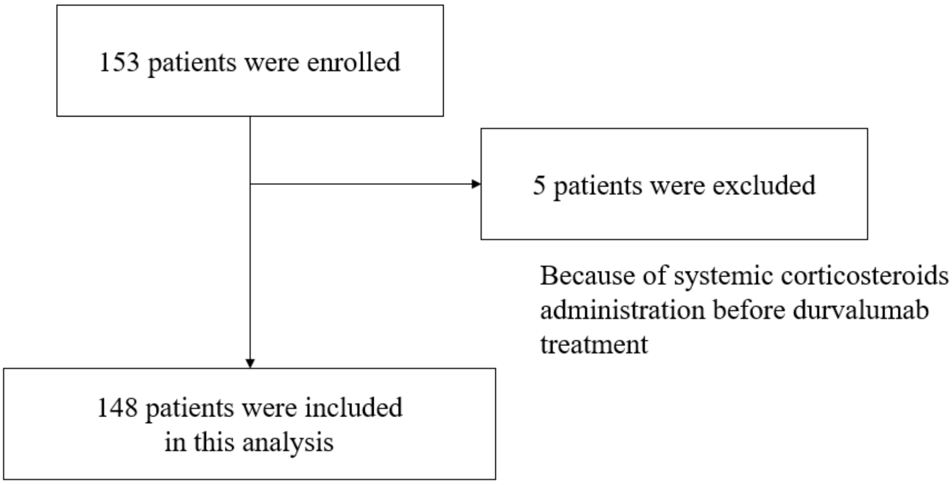

Supplementary Figure 3. CT images of grade 3 ILD in a patient with ILAs.

On the CT findings before durvalumab treatment (A, B), ground glass attenuation in ILAs (arrowheads) was detected in the peripheral fields of both lung on CT (C). Grade 3 ILD occurred six weeks after durvalumab treatment (D, E).

CT, computed tomography; ILD, interstitial lung disease; ILAs, interstitial lung abnormalities

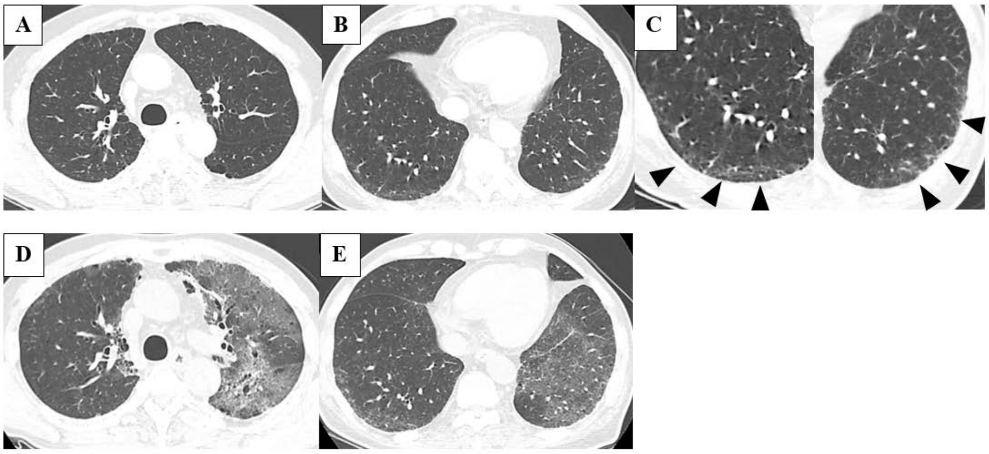

Supplement: Supplementary file 1 [file cancers-14-06236-s001.zip › cancers-2054060-supplementary.pdf]
